# Identifying temporal variation in reported births, deaths and movements of cattle in Britain

**DOI:** 10.1186/1746-6148-2-11

**Published:** 2006-03-30

**Authors:** Susan E Robinson, Rob M Christley

**Affiliations:** 1 Epidemiology Group, Department of Veterinary Clinical Sciences, University of Liverpool, Leahurst, Neston, South Wirral, UK

## Abstract

**Background:**

The accuracy of predicting disease occurrence using epidemic models relies on an understanding of the system or population under investigation. At the time of the Foot and Mouth disease (FMD) outbreak of 2001, there were limited reports in the literature as to the cattle population structure in Britain. In this paper we examine the temporal patterns of cattle births, deaths, imports and movements occurring within Britain, reported to the Department for the Environment, Food and Rural Affairs (DEFRA) through the British Cattle Movement service (BCMS) during the period 1^st ^January 2002 to 28^th ^February 2005.

**Results:**

In Britain, the number of reported cattle births exhibit strong seasonality characterised by a large spring peak followed by a smaller autumn peak. Other event types also exhibit strong seasonal trends; both the reported number of cattle slaughtered and "on-farm" cattle deaths increase during the final part of the year. After allowing for seasonal components by smoothing the data, we illustrate that there is very little remaining non-seasonal trend in the number of cattle births, "on-farm" deaths, slaughterhouse deaths, on- and off-movements. However after allowing for seasonal fluctuations the number of cattle imports has been decreasing since 2002. Reporting of movements, births and deaths was more frequent on certain days of the week. For instance, greater numbers of cattle were slaughtered on Tuesdays, Wednesdays and Thursdays. Evidence for digit preference was found in the reporting of births and "on-farm" deaths with particular bias towards over reporting on the 1^st^, 10^th ^and 20^th ^of each month.

**Conclusion:**

This study provides insight into the population and movement dynamics of the British cattle population. Although the population is in constant flux, seasonal and long term trends can be identified in the number of reported births, deaths and movements of cattle. Incorporating this temporal variation in epidemic disease modelling may result in more accurate model predictions and may usefully inform future surveillance strategies.

## Background

Mathematical modelling approaches are increasingly being employed to inform disease control strategies. Interest in these techniques in this context has been greatly augmented by recent disease outbreaks within the British cattle population. The accuracy of such models relies upon accurate estimates of population structure as temporal trends in the births, deaths and movements of cattle may impact substantially on pathogen transmission dynamics. For instance, birth rate may affect rapidity of spread due to supply of susceptible individuals into the population. Understanding the dynamics of the cattle population may also inform timing of resources and therefore the efficacy of surveillance schemes.

In addition to factors associated with pathogen transmissibility and host susceptibility, population characteristics can drive the temporal and spatial patterns of disease occurrence. For example, the widespread movement of livestock (often over considerable distances) that occurred prior to the detection of foot and mouth disease (FMD) in 2001 resulted in transmission of disease to several spatially distinct foci, one of the main differences between the outbreak of 2001 compared with that of 1967 [1]. An association between movements of infected cattle and the observed geographical pattern of disease has also recently been shown for bovine tuberculosis by Gilbert *et al*. [2]. Although reports of temporal characteristics in cattle movements in Britain already exist in the literature [3], quantification and additional exploratory analysis of the extensive data is required.

The individual identification and tracing of the cattle population is a requirement of all member states of the European Union (Regulation 820/97). Within Britain a centralised tracing system capable of identifying individual cattle for the purposes of public health was established in the form of the British Cattle Movement Service (BCMS). Mitchell et al. (2005) provided a short historical overview of the changes to the reporting of cattle movements in Britain. Since 2001 it has been mandatory for all keepers of cattle in Britain to register births, deaths and movements to BCMS via telephone, post or internet. This data is then collated by the Department for Environment, Food and Rural Affairs (DEFRA) as part of the Rapid Analysis and Detection of Animal Risk (RADAR) information management system [4].

This extensive database resource provides the reported event (births, deaths and movement) histories of all cattle in Britain. The database of cattle births, deaths and movements reported to BCMS contains records from as early as 1996. However, initially, data reporting by animal keepers was not mandatory. Also, as a result of the Foot and Mouth Disease outbreak of 2001, many animals were slaughtered and movement restrictions were imposed on the industry, and data collected during this period are atypical. In this paper we consider only movements of cattle for the period 1^st ^Jan 2002 to 28^th ^Feb 2005.

Time series analysis, of which data smoothing forms a necessary first step, can be applied to temporal data to identify two basic components: trend and seasonality [5,6]. Trend represents a general systematic linear or nonlinear component that changes over time and does not repeat. Seasonality represents trend that repeats itself in systematic intervals over time. Identifying these two components in time series data can help to understand underlying processes and also to predict future trends. In this paper we aim to identify long term trends, as well as seasonality in cattle population dynamics and cattle movements within Britain by analysing data on cattle movements collected by the BCMS and supplied through DEFRA's RADAR information system.

## Results

### Time series traces

For each type of event (birth, "on-farm" death, slaughterhouse death, import and on-movements) the raw data, 3-point moving average, residuals (after accounting for the 3-point moving average) and 53-point weighted moving average are illustrated.

**Figure 1:**
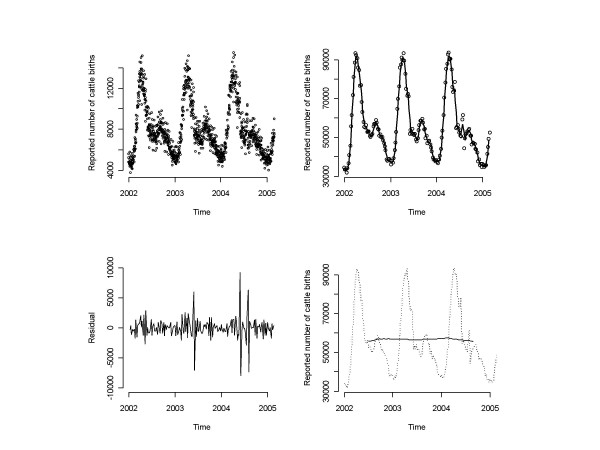
**Time series for cattle births reported to BCMS over the period 1^st ^Jan 2002 and 28^th ^Feb 2005**. (a) Raw data plotted at daily intervals (b) 3-point moving average (raw weekly data shown as unconnected symbols) (c) Residuals after accounting for the 3-point moving average (d) 53-point moving average (raw weekly data shown as unconnected symbols).

Figure 1 shows raw (daily observations) and smoothed (weekly observations) data for reported cattle births in Britain from 1/1/2002 to 28/2/2005 inclusive. The series in Figure 1a and 1b suggests a very regular repeating pattern comprising an annual spring peak (approximately 90,000 reports of births per week) and a trough at the end of each year (approximately 35,000 reports of births per week). Each annual cycle exhibits strong seasonal fluctuations with peaks in the spring and secondary smaller peaks occurring in the autumn. Smoothing over a 3 week period emphasizes the major features of the data, notably the seasonal pattern further (Figure 1b). Plotting of the residuals after accounting for the 3-point moving average (raw data at time *t*, minus the 3-point moving average at time *t*) shows a greater degree of variation in the early summer of 2003 and also two further periods during the summer of 2004 (Figure 1c). After accounting for the seasonal component in reported births, there appeared to be limited residual variation in the data (often termed noise) and no marked change in the number of births during the years 2002–2004 (Figure 1d).

**Figure 2:**
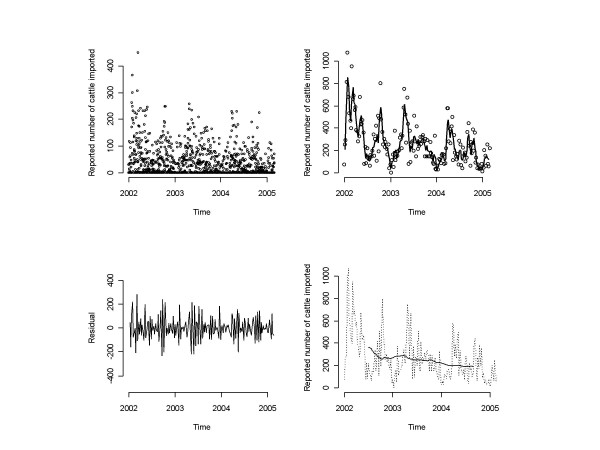
**Time series for cattle imports reported to BCMS over the period 1^st ^Jan 2002 and 28^th ^Feb 2005**. (a) Raw data plotted at daily intervals (b) 3-point moving average (raw weekly data shown as unconnected symbols) (c) Residuals after accounting for the 3-point moving average (d) 53-point moving average (raw weekly data shown as unconnected symbols).

There was no clear trend in live cattle imports evident from the raw import data (Figure 2a). However, on close inspection of the trace, seasonal fluctuations are discernable which upon smoothing (Figure 2b) become more evident. However, peaks in imports appear at different times during successive years. In 2003 a strong spring peak in the number of imported animals occurred whereas two smaller peaks in spring and autumn occurred in 2004. Plotting of the residuals reveals relatively constant noise over time, but a small systematic reduction in residual variance over time may be evident (Figure 2c). Smoothing of the data to reduce the effect of seasonality in the data (53-point weighted moving average) demonstrates a generally decreasing trend in the number of cattle imports since 2002 (Figure 2d).

**Figure 3:**
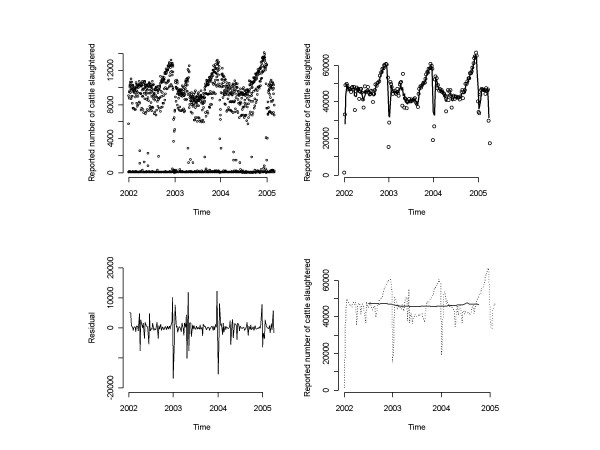
**Time series for cattle slaughter reported to BCMS over the period 1^st ^Jan 2002 and 28^th ^Feb 2005**. (a) Raw data plotted at daily intervals (b) 3-point moving average (raw weekly data shown as unconnected symbols) (c) Residuals after accounting for the 3-point moving average (d) 53-point moving average (raw weekly data shown as unconnected symbols).

Figure 3a suggests that deaths reported at slaughterhouses increase during the latter months of the year prior to a sharp decline associated with the last few of weeks of the year. Figure 3b emphasizes the seasonal pattern and reduces the effect of daily fluctuations which feature in the time plot of the raw data. Several important local features of the data are discernable from the time series plots. In Figure 3a a second trace of lower magnitude can be identified which is attributable to the reduced numbers of cattle slaughtered at the weekend. In Figure 3b there a sharp decrease in the number of cattle slaughtered towards the end of each year associated with closure of many slaughterhouses around Christmas and New Year. The plot of residuals is dominated by large spikes associated with the Christmas period, 2002 and 2003 (explained by the extreme effect of Christmas week), and spring 2003 (Figure 3c). The residual spike associated with the Christmas period of 2004 was not as large as that in the preceding years. This may be partly attributable to the Christmas of 2004 falling on Saturday and Sunday, which are routinely identified as days on which fewer numbers of cattle are slaughtered but it may also be that data are incomplete. Within each year slaughterhouse deaths appear to be relatively constant during the period 2002 to 2004 (Figure 3d).

**Figure 4:**
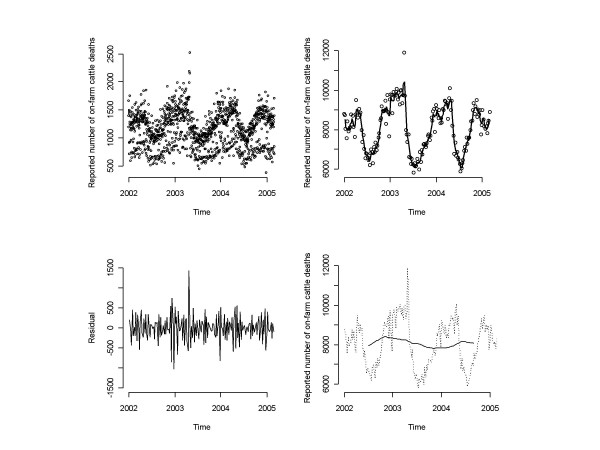
**Time series for "on-farm" cattle deaths reported to BCMS over the period 1^st ^Jan 2002 and 28^th ^Feb 2005**. (a) Raw data plotted at daily intervals (b) 3-point moving average (raw weekly data shown as unconnected symbols) (c) Residuals after accounting for the 3-point moving average (d) 53-point moving average (raw weekly data shown as unconnected symbols).

Figure 4 features the temporal plots for "on-farm" animal deaths. It is possible to identify a strong seasonal component in both Figures 4a and 4b with peaks during the winter months and troughs in the summer months. As is the case for several other event types, in Figure 4a a second trace of lower magnitude but similar cyclical pattern is present. This is attributable to the reduced number of cattle deaths reported to have occurred at the weekend. After allowing for the seasonal component there appears to be low residual variation in deaths other than what would normally be deemed as noise. However there are spikes during the early few months of 2003 that are residual to what can be explained by the seasonality. Year on year there appear to be small fluctuations in "on-farm" deaths (Figure 4d).

**Figure 5:**
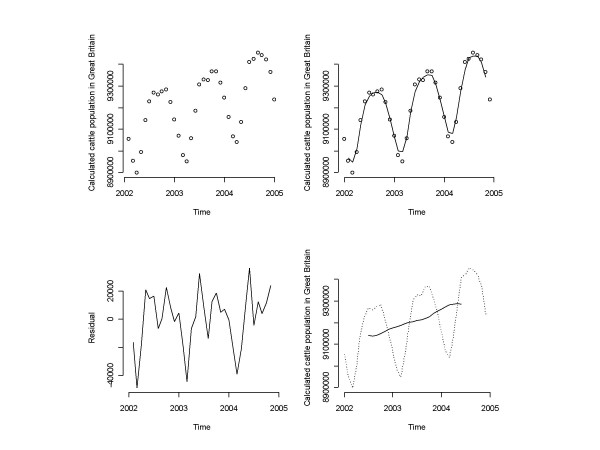
**Time series for the calculated cattle population in Britain per month reported by BCMS over the period 1^st ^Jan 2002 and 31^st ^Dec 2004**. (a) Raw monthly data (b) 3-point moving average (raw data shown as unconnected symbols) (c) Residuals after accounting for the 3-point moving average (d) 13-point moving average (raw monthly data shown as unconnected symbols).

Figure 5 represents the monthly data for the calculated cattle population in Britain. Within each year the cattle population peaks during the spring, summer and autumn months. As both slaughterhouse and "on-farm" cattle deaths increase towards the end of the year (Figures 3 and 4), so the cattle population decreases. After accounting for this seasonal flux, the cattle population in Britain appears to have been steadily increasing during recent years.

**Figure 6:**
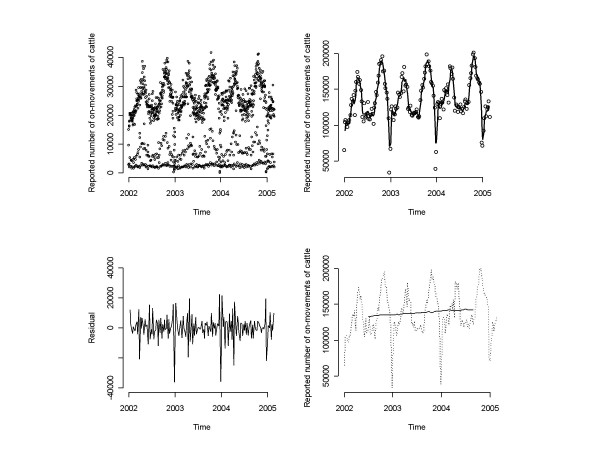
**Time series for "on" movements of cattle reported to BCMS over the period 1^st ^Jan 2002 and 28^th ^Feb 2005**. (a) Raw data plotted at daily intervals (b) 3-point moving average (raw weekly data shown as unconnected symbols) (c) Residuals after accounting for the 3-point moving average (d) 53-point moving average (raw weekly data shown as unconnected symbols).

The data for the reported on-movements of cattle onto all types of premises are plotted in Figure 6. Although we have not presented the results for off-movements of cattle from premises, the data are very similar and the traces appear to replicate those seen for the on-movements. Therefore all of the results described below for trends in on-movements also apply to those of off-movements, as is expected due to the dual-reporting of cattle transferred between two premises; if an animal arrives on one premise (an on-movement reported) it must have left another (an off-movement reported). In Figure 6a, the reporting of on-movements of cattle exhibit strong temporal cycles associated with spring and autumn peaks, of which, the autumn peak is the larger (this is more apparent after smoothing represented in Figure 6b). Additional traces of similar trend but of smaller magnitude were observed with both on- and off-movements, reflecting the reduced number of movements occurring on Saturday and, particularly on Sundays (Figure 6a). Plotting the residuals does not highlight any further temporal trends. The 53-point weighted moving average indicates that on-movements have been steadily, but consistently increasing over the study period (Figure 6d).

As well as examining the number of cattle moving according to day of the year, movements were also grouped by animal holding premises and the number of premises reporting births, deaths or movements on any given day was analysed. The temporal trends for the number of farms reporting animal births, imports, deaths and on and off movements for each day appeared to be very similar to the traces for the number of animals of each event respectively, therefore suggesting that the average size of batch movements of animals at different times of the year do not vary greatly. The majority of farms reported singular occurrences of births and deaths.

### Modelling of calendar effects

**Figure 7:**
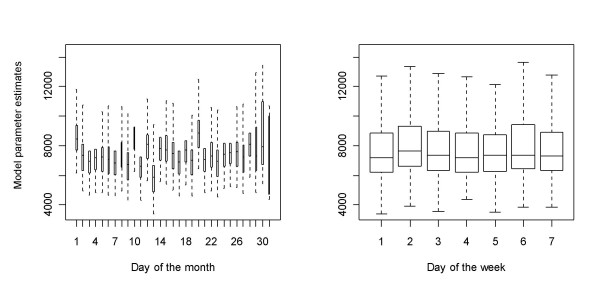
**Box plots of the fitted model outputs for the generalised linear model for cattle births reported to BCMS over the period 1^st ^Jan 2002 and 28^th ^Feb 2005**. (a) effect of day of the month (b) effect of day of the week (1- Sunday : 7-Saturday). Boxes incorporate the standard errors around the parameter estimate with error bars indicating the 95% confidence interval.

**Figure 8:**
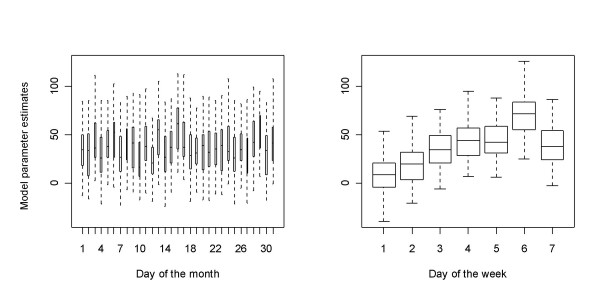
**Box plots of the fitted model outputs for the generalised linear model for live cattle imports reported to BCMS over the period 1^st ^Jan 2002 and 28^th ^Feb 2005**. (a) effect of day of the month (b) effect of day of the week (1- Sunday : 7-Saturday). Boxes incorporate the standard errors around the parameter estimate with error bars indicating the 95% confidence interval.

**Figure 9:**
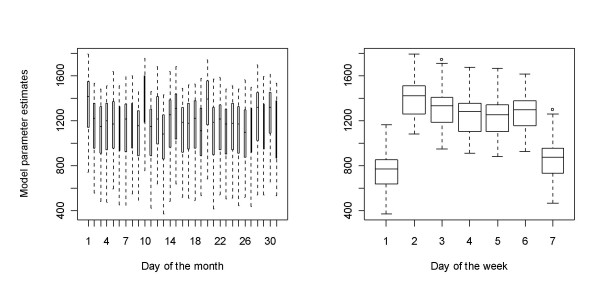
**Box plots of the fitted model outputs for the generalised linear model for "on-farm" cattle deaths reported to BCMS over the period 1^st ^Jan 2002 and 28^th ^Feb 2005**. (a) effect of day of the month (b) effect of day of the week (1- Sunday : 7-Saturday). Boxes incorporate the standard errors around the parameter estimate with error bars indicating the 95% confidence interval.

Generalised linear models were used to examine and compare the different effects of the day of the week, the day of the month, month and year on the number of reported events. When fitted in the same model with day of the week and day of the month, the associations between the variables year and month on reporting of events (births, imports, "on-farm" and slaughterhouse deaths and movements) were in agreement with the trends reported above and shall therefore not be presented for a second time. The model parameter values and standard errors are plotted for births, imports and "on-farm" deaths in Figures 7, 8 and 9, respectively. For all cattle events, as well as significant effects of month and year (P < 0.0001), the day of week and the day of month were also significantly associated with the numbers of reported events (P < 0.001).

Figure 7 demonstrates the increased reporting of calf births on several days of the month; particularly the 1^st^, 10^th^, 20^th^, 28^th ^and 30^th^. From the model the dates 1^st^, 10^th ^and 20^th ^were all associated with a significantly (P < 0.0001) increased number of reported births on these dates even after allowing for month and day of the week in the model. Calf births were less likely to be reported as having occurred on Sundays and were more likely to be reported as having occurred on Mondays (a significant association; P < 0.002) and Fridays compared to other days of the week.

**Figure 10:**
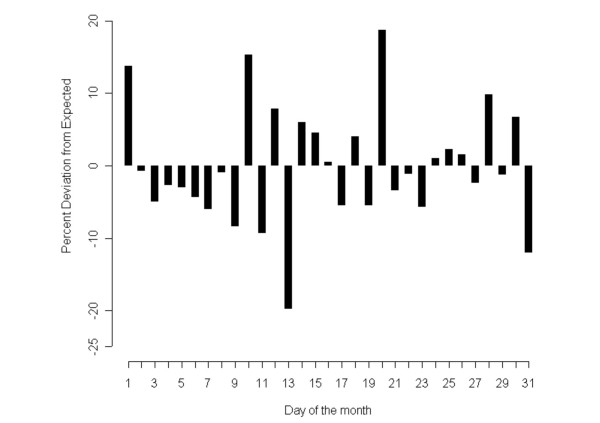
Digit preference for recall of cattle births using reported birth dates to BCMS and the percent of deviation from expected by day of the month after accounting for the different number of days in each month.

After accounting for months with fewer than 31 days, further exploration of the data on reported calf birth dates indicated a notable tendency to report the 1^st^, 10^th ^and 20^th ^of the month. There were deficits in the reports for odd numbers adjacent to multiples of 10, such as the 9^th^, 11^th^, 19^th ^and 31^st ^of each month (Figure 10). There also a clear deficit in births reported to be on the 13^th ^of any month with over 20% fewer births reported on this day compared with the number expected. A similar pattern was also reported for "on-farm" deaths but was not evident for other events, (i.e. imports, slaughterhouse deaths and on- or off-movements (data not shown)).

The generalised linear model output for live cattle imports (Figure 8) revealed a significantly increased association between the 16^th ^of any month and the number of cattle imported on that day when compared to the 1^st^. There was a trend for increased number of import movements occurring later in the week and Friday was significantly (P < 0.001) associated with more import movements compared to Mondays and Sundays.

The first four days and the 24^th^–27^th ^and 30^th ^and 31^st ^days of any given month were associated with significantly (P < 0.05) fewer cattle slaughtered on these days. Mid- periods of any given month were associated with an increased number of cattle reportedly slaughtered. Not surprisingly, Tuesdays, Wednesdays and Thursdays were associated with significantly more slaughterhouse deaths when compared to Mondays and Fridays (P < 0.001), which were also significantly associated with increased numbers of deaths compared to weekend days (P < 0.001).

Figure 9 illustrates the significantly (P < 0.001) increased number of reported "on-farm" deaths occurring on the 1^st^, 10^th ^and 20^th ^of the month, as highlighted above. "On-farm" deaths reportedly occurring on a Monday were overrepresented compared to other days of the week and this effect was found to be significant (P < 0.001) compared to all others days of the week. Weekdays were also significantly more associated with reported "on-farm" deaths than were weekend days.

## Discussion

In this paper we have identified seasonal and other temporal trends in the reported births, deaths and movement of cattle within Britain. The findings reported in this paper are supported by Mitchell *et al*. [3]. However, our analysis has taken a more rigorous approach to the temporal structure of the data: extracting temporal trends in the residuals unexplained by the seasonality in the data and extracting long term trends whilst allowing for seasonality in the data. The time period examined in this paper is also more recent and does not include the movements occurring during the UK FMD outbreak of 2001, which was a period of unusual cattle movement patterns due to the implementation of disease control measures.

Cattle are managed on animal holdings, with management often determined by season. Hence it was not surprising that we found considerable variation, with distinct seasonality in the number of reported births (reflecting the spring clustering in calvings), deaths and movement of cattle. Trends in the dynamics of the cattle population, (i.e. births and deaths) indicate strong seasonal fluctuations accompanied by relatively small changes year on year. There is evidence that the cattle population is steadily increasing year on year, which concurs with the birth rate in recent years exceeding the overall death rate. However, the effect of gradual restocking of farms following the FMD outbreak in 2001 and improvements in data capture and data quality over recent years are likely to have contributed to the observed increase in the number of cattle in the population.

An important feature of this analysis is the examination of residuals in the temporal data after allowing for seasonal fluctuations. The residuals for live imports of cattle reveal no obvious trend and can be regarded as noise (unexplained variation). However for most other events, examination of the residuals reveals further temporal features in the data. For births, slaughterhouse and "on-farm" deaths a spring spike in 2003 is evident that is not explained by seasonal extremes in trends. This observation can not be explained by changes in data management or quality by BCMS at that time (Mr A. Pryor, personal communication,). Although not necessarily related, the spring spike in residuals in 2003 does coincide with a change in the legislation governing cattle movements when the stand-still rule in England and Wales reduced from 20 to 6 days. Other biases may account for the observation, and further exploration may be warranted.

By examining the appearance of spikes on the residual plots in this way, outbreaks of disease leading to higher mortality may be highlighted. However, outbreaks of disease are often localised and therefore analysing regional, as opposed to national data, may be more informative. Furthermore, highlighting periods in real-time when the number of "on-farm" deaths are above or below the normal seasonal fluctuations, may lead to a more reactive and flexible surveillance. This approach to identifying localised disease "hot spots" has been discussed on a small spatial scale for cases of gastrointestinal disease in humans [7]. The reporting of movements to BCMS is, however, not available in real time and therefore, in terms of real time surveillance, it is not conceivable that this approach could be taken at present.

In this paper we characterised the data regarding cattle deaths into "on-farm" deaths, reported by agricultural holdings, landless keepers, knackers yard, hunt kennels, markets and on common land (perceived to be culled or diseased cattle) and those occurring at slaughterhouse premises (assumed to be entering the food or animal feed chain). Although this distinction has proved useful the assumption that cattle arriving at slaughterhouses enter the food or feed chain may not be the case for a small proportion of cattle that arrive at the slaughterhouse. Equally, our assumption that cattle deaths on agricultural holdings, at hunt kennels, or knackers yards etc., are due to disease is also likely to be an overestimation of the amount of disease as many cattle on farms will be culled due to age related factors and may not be diseased. Therefore the data for "on-farm" deaths may be more useful in disease surveillance if additional information collected.

Importation of live cattle also affects the cattle population dynamics within Britain. In contrast to the trend for births and deaths, imports have been decreasing year on year since 2002, possibly reflecting the increased demand for cattle immediately following the 2001 FMD outbreak. This reduction in the numbers of cattle imported from other countries may have important implications for risk assessments associated with the importation of cattle disease into the national herd. In addition other temporal trends highlighted such as periods of the year, dates of the month and days of the week when increased numbers of cattle are imported could help to direct resources as part of an informed surveillance program.

We have presented evidence that records of cattle births and "on-farm" deaths taken from the RADAR information management system are subject to a reporting bias, namely digit preference, with preferential reporting of dates ending in a multiple of 10's, even numbers or the first of the month. It is unlikely that there would be any biological explanation for this effect. Digit preference, the preferential reporting of dates or numbers by subjects, typically those ending in zero or five, is a well documented reporting bias that has been investigated in several health-related contexts, including blood-pressure measurements [8], self-reported height and weight [9] and date of onset of last menstrual cycle [10]. The evidence that this form of bias appears only in the reporting of births and "on-farm" deaths may be due to several factors at the animal holding level. Firstly, as births and deaths only occur on one animal holding, it is their sole responsibility to report. There is no method of cross-checking the date as is the case for movements of cattle off and onto premises which involves both parties reporting the movement. Secondly, different rules exist for the reporting of different events. Calf births in Britain must be reported to BCMS by animal keepers within 27 days, deaths must be reported within 7 days, whereas movements of cattle must be reported within 36 hours of the movement occurring. Hence, there may be differential recall error for different events due to the variation in intervals permitted between event(s) and reporting. It would be of interest to explore, in consultation with animal keepers, the reasons for this bias. For instance, the method (post, telephone or internet) by which births are reported to BCMS may affect the degree of bias present.

As well as error occurring in reporting, others sources of error may be introduced during data editing. Within the raw data reported by animal keepers obvious and illogical discrepancies exist, e.g. the reporting of a birth date that is after death has been reported to occur. In such cases, data editing by data suppliers (either BCMS and/or DEFRA) is undertaken to ensure that events in cattle movement histories are logical and sequential. Hence, this editing process may also be a source for the preferential selection of particular dates.

The apparent preferential reporting of Mondays as the day on which most births and "on-farm" deaths occur on animal holdings may result from biased observation. Due to the (anecdotally) lower intensity of observation of cattle during the weekend, some births and "on-farm" deaths that occur over a weekend may only be detected on Mondays when closer inspection of the cattle herd resumes. It may also be the case that if cattle fall ill during the weekend, euthanasia by a veterinary surgeon would not occur until after the weekend, when consultation charges may be lower.

It is likely and, indeed, intended that data on cattle movements obtained via the RADAR information management system will become more widely available to scientific researchers in the future. It is important that biases inherent within the database be considered. In general terms, the digit preference reporting bias that we have identified may only cause small discrepancies between the reported and the actual dates of calf births and deaths. Although for many studies this may not be important, some studies may need to consider and adjust for the effect of this bias; for example studies assessing mortality in calf cohorts using data extracted from RADAR information management system, where small deviations in the age of calves are likely to be important. Methods exist for the correction of this bias within datasets [11]. However it will often be the case that an awareness of the bias may be all that is required.

Whilst digit preference is a natural phenomenon associated with recall, measures to reduce or avoid this source of bias within the data may be worthwhile. Consultation with animal keepers may suggest important improvements for the methods of reporting of births and deaths that may lead to a reduction in the bias. Identification of animal holdings for whom records suggest substantial digit preference may be used to target incentives to improve the accuracy of reporting births and deaths. It is also of significance to highlight that in different applications, evidence of bias within data, can also aid in the detection of fraudulent claims [12].

A limitation of this study is the assumption that all movements, births and deaths of cattle are subsequently reported to DEFRA. There has been some speculation within the industry as to the extent of unreported or misreported cattle movements and therefore the efficacy of surveillance policy based on incorrect field data. Further issues of data quality associated with the data handling may also be introducing sources of error.

## Conclusion

The recording of sequential observations in the form of daily cattle births, deaths and movements within Britain provides a large data set, archetypal for use in time series analysis. Identifying trends in movement of cattle and the underlying population dynamics may assist the planning of appropriate disease surveillance schemes that can be seasonally adjusted to cope with increasing surveillance at times of the year when movements are at a peak. Further time series analysis may also aid in the prediction of future trends in movements and population dynamics. Using complex time series analysis the ability to forecast and predict future number of movements that may occur on a particular day may of use again for surveillance purposes. However, restructuring within the cattle industry, in response to rapid changes in government legislation (lifting of the ban on cattle over 30 months entering the food and feed chain), are also likely to cause continued changes to the cattle population structure in Britain. These changes require continued monitoring.

## Methods

### Data source

All data regarding cattle births, deaths and movements including imports were obtained from DEFRA's RADAR information management system based on data downloaded from the BCMS cattle tracing system (CTS) on 08/04/2005.

A dataset containing records of cattle births and deaths since 1996 (coinciding with the introduction of cattle passports) was extracted. Initial graphical exploration of the data indicated that records (and in particular on and off movements) pre 2001 were incomplete. Changes in recording methods, scrutiny and capture, as well as the FMD outbreak of 2001 have all contributed to this period of uncertainty and variability in the dataset. Therefore all further analyses were conducted on reported births, deaths and movements of cattle occurring between 1/1/2002 and 28/2/2005.

The original database contains records of births, deaths, "on" and "off" movements and imports reported by each animal holding for any given day. From this we created a dataset including date of event (an event being a birth, death or movement), location type and number of animals involved in the event. In the original database an "off" or "on" movement may be classified into several further classifications depending on the source of the information regarding the movement. For our analyses we collapsed these subdivisions to define the movement only as an on- or off movement of cattle.

Using information from the location type descriptor, where possible (approximately 80% of records), we differentiated between cattle deaths for the purposes of food or animal feed purposes (assumed to be deaths reported on slaughterhouse premises) and deaths due to culling or disease (assumed to be deaths reported by animal holdings not associated with the slaughter of cattle; agricultural holdings, landless keepers, common land, knackers yards, markets and hunt kennels). For the purposes of this paper these shall be defined as "on-farm" deaths. Deaths at other location types were contained within the data, however this only accounted for a very small percentage (<1%) of the reported deaths and were therefore not included in this analysis.

The count of cattle on each animal holding on the 1^st ^of every month was also available in the data extract from DEFRA's RADAR information management system. Summing across animal holdings allowed the total cattle population in Britain to be calculated. Data storage, management and manipulation was achieved using a number of software packages including PostgreSQL [13], Microsoft Access and Excel [14].

The choice of time interval for representing the data is important; concise data sets are more readily manipulated but important information in the data may be lost if long intervals are chosen. The data on births, deaths and movements exist as daily observations. Initially, we plotted the raw daily data but when smoothing the data we summed across weeks and also by months. After initial investigation, summing monthly data resulted in essential features of the original trace being lost. Treating the data as weekly observations appeared to be a suitable compromise and intuitive due to the 6-day movement restriction that applies to farms in England and Wales. This restriction prohibits any movement of livestock off an animal premise if livestock have been moved onto the premises in the last 6 days.

### Data smoothing

Temporal trends evident after plotting the raw data, were further explored using a range of smoothing techniques; a useful way of making seasonal components clearer. Here we used moving average smoothing which replaces each element of the series by the mean of *n *surrounding elements, where *n *is the width of the smoothing window [5,6]. Essentially, taking moving averages involves running a moving window over the data and taking averages of points falling within each of these windows. This has the advantage of highlighting broad patterns by removing localised fluctuations, often termed as noise. For this analysis we used weekly data with windows of 3-point (week) and 53-point (week) intervals. Three point moving averages of the data were taken to highlight seasonal effects. To dampen the effect of seasonality and highlight possible long-term trends, we smoothed the data by taking a 53-point weighted moving average. The contribution of each week was weighted to allow for the fact that the same week of the year (n ± 26) appears twice in the data window. Observing the residuals after subtracting the 3-point moving average from the data also allowed examination of local fluctuations in the data. For the smoothing of the cattle population data, (monthly observations) we smoothed the data by taking 3-point and 13-point (weighted) moving averages. All exploratory analysis of time series data was generated in the statistical software package R [15].

### Generalised linear modelling

To explore the relationship between the number of births, deaths and movements of cattle and time varying covariates we fitted generalised linear models (GLM) using the daily records as the independent variable with year, month, day of the month, and day of the week included as dependent variables [16]. As many of the counts of events were very large, the models rely upon asymptotic normal distribution approximations using a linear regression model. Comparisons between models using Poisson and normal distribution approximations did not alter the inference of the results even where counts were smaller, such as for the import data. All GLM were run in the software package R. Residuals were examined for evidence of departure from normality, which might signify model inadequacies.

Where the GLM output suggested potential digit preference this was further evaluated by calculating the expected distribution of births per days of the month, taking into account the number of months with less than 31 days, and comparing the observed number to the expected number of days. Our assumption was that over a sufficiently long period of time, births occur randomly over time.

## Authors' contributions

SE carried out all descriptive and statistical analysis. RC conceived of the study provided guidance and advice on all aspects of analysis and writing.
